# Onset of action for loratadine tablets for the symptomatic control of seasonal allergic rhinitis in adults challenged with ragweed pollen in the Environmental Exposure Unit: a post hoc analysis of total symptom score

**DOI:** 10.1186/s13223-017-0227-4

**Published:** 2018-01-16

**Authors:** Mark W. Tenn, Lisa M. Steacy, Charlene C. Ng, Anne K. Ellis

**Affiliations:** 1 Department of Biomedical and Molecular Sciences, Queen’s University, Kingston, ON Canada; 2 Allergy Research Unit, Kingston General Hospital, 76 Stuart Street, Kingston, ON Canada; 3 Division of Allergy & Immunology, Department of Medicine, Queen’s University, Kingston, ON K7L 2V7 Canada; 4 Bayer U.S. LLC, Whippany, NJ USA

**Keywords:** Allergic rhinitis, Environmental Exposure Unit, Loratadine, Onset of action, Ragweed pollen, Seasonal allergies, Outdoor allergy

## Abstract

**Background:**

Loratadine is a second-generation, non-sedating antihistamine used for the relief of allergic rhinitis symptoms. Previous studies reported that when loratadine was encapsulated, the onset of action for symptom relief was 180 min. However, unmodified loratadine tablets were not evaluated at that time. Using data from a previously published Environmental Exposure Unit (EEU) study comparing azelastine nasal spray with loratadine tablets, cetirizine tablets, and placebo, this post hoc analysis determines the onset of action of loratadine tablets (i.e. unmodified) by analyzing the total symptom score for the relief of nasal and ocular seasonal allergic rhinitis (SAR) symptoms.

**Methods:**

A Phase IV, randomized, single-center, double-blind, placebo-controlled, double-dummy, four-way crossover study was conducted in the EEU. Seventy participants were randomized sequentially into one of the four treatments during ragweed pollen exposure. Nasal and ocular symptom scores were self-reported by the participants and recorded. The original study analysis was carried out by evaluating the nasal symptom scores only. For this post hoc analysis, both nasal and ocular data from the loratadine and placebo treatment arms were analyzed. The primary endpoint for this analysis was the onset of action of loratadine as measured by the change in total symptom score (TSS) from baseline in comparison to placebo. The onset of ocular symptom relief using the total ocular symptom score (TOSS) was also reported.

**Results:**

Loratadine tablets demonstrated a significant and durable improvement in both TSS (*P* = .005) and TOSS (*P* = .013) at 75 min post-treatment administration compared to placebo. The mean proportion of participants reporting none or mild for all component symptoms of TSS and TOSS at 75 min and thereafter was significantly higher in the loratadine (TSS, *P* = .0005; TOSS, *P* ≤ .0001) vs. placebo treatment arm.

**Conclusions:**

The onset of action of loratadine tablets was 75 min for the relief of nasal and ocular symptoms in adults with SAR. These results suggest a faster onset of action for loratadine tablets (75 min) compared to previously reported studies which were conducted with modified (i.e. gelatin-encapsulated) loratadine tablets (180 min).

*Trial registration* Clinicaltrials.gov identifier NCT00561717

**Electronic supplementary material:**

The online version of this article (10.1186/s13223-017-0227-4) contains supplementary material, which is available to authorized users.

## Background

Allergic rhinitis (AR) is an inflammatory upper respiratory disorder involving IgE-mediated inflammation of the nasal mucosa that affects approximately 10–30% of the world population [1–3]. Individuals with seasonal allergic rhinitis (SAR) can be reactive to several types of pollen allergens (such as those from ragweed, grass, and tree pollens), experience symptoms such as sneezing, nasal congestion, nasal and nasopharyngeal itching, and have ocular symptoms like red/itchy and watery eyes [4]. Oral antihistamines are the first line treatment for SAR, of which second generation agents are preferred due to fewer sedative effects, and a lack of impairment of cognitive function compared to first generation compounds [5, 6].

Loratadine is a second generation oral histamine H_1_-receptor antagonist [7]. Previous studies have demonstrated its efficacy for the relief of SAR symptoms over placebo [8, 9]. However, the onset of action for loratadine has been variably reported, ranging from 75 to 180 min [10–15].

As defined per the US FDA draft guidance for the clinical development programs of AR drug products, the onset of action is the first time point after start of treatment when the product demonstrates a statistically significant change from baseline in the primary efficacy endpoint that is greater than placebo and is durable throughout the dosing period [16]. Since 2000, the FDA recognized the Environmental Exposure Unit (EEU) as one of three methods to evaluate the onset of action of products for AR treatment [16]. In comparison to natural allergen exposure studies, controlled allergen challenge facilities such as the EEU are internationally recognized and clinically validated models of AR that can be used to evaluate products intended for AR treatment [17]. The EEU allows for the simultaneous exposure of a controlled quantity of allergen to large groups of study participants. Confounding environmental variables such as humidity, air quality, CO_2_ levels, and temperature can be tightly controlled, creating an environment suitable for the evaluation of novel anti-allergic medication [17–19].

A previously reported EEU study evaluated the onset of a topically applied intranasal antihistamine spray for the improvement of SAR symptoms, and loratadine was included as one of the comparator treatments [10]. In that study, an onset of action of 75 min for loratadine was reported based on the evaluation of nasal symptoms alone [10]. Two prior EEU studies conducted by Day et al. [12, 13] suggested a longer onset of approximately 180 min for loratadine. However, the authors evaluated the onset of action of a commercially available loratadine tablet they had modified by encapsulating with gelatin. The authors did not demonstrate bioequivalence of the encapsulated form to the currently available marketed, non-encapsulated tablet form. The aim of the current study was to conduct a post hoc statistical analysis using data from the Ellis et al. [10] study to determine the onset of action of unmodified loratadine tablets in the EEU.

## Methods

### Study design and treatment

The clinical methodology of the trial has been published elsewhere [10]. In brief, the trial was a Phase IV, randomized, single-center, double-blind, placebo-controlled, double-dummy, four-way crossover study (clinicaltrials.gov identifier NCT00561717). The study was reviewed and granted ethical clearance by the Queen’s University Health Sciences and Affiliated Teaching Hospital Research Ethics Board. It was conducted in accordance with the Declaration of Helsinki (Somerset West Amendment 1996) and the International Council for Harmonisation Guideline on Good Clinical Practice.

**Fig. 1:**
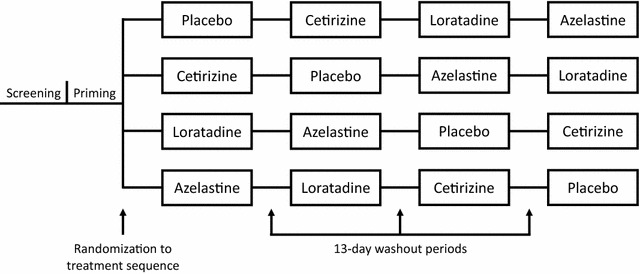
Study diagram

The study was conducted in the EEU and was comprised of a screening visit, a priming period, and four dosing/exposure periods with a 13-day washout between periods (Fig. 1). After eligibility was determined, qualifying participants were randomized to a treatment sequence comprised of one dose of each of the four study medications—azelastine nasal spray, loratadine tablet, cetirizine tablet, and placebo. All study treatments were administered as a combination of an oral tablet (active or placebo) and nasal spray (active or placebo) to maintain study blinding.

Each dosing period consisted of an 8-h ragweed pollen challenge in the EEU (mean pollen levels of 3500 ± 500 grains/m^3^). The level of pollen used is consistent with other EEU studies used to determine the onset of allergy products and provides consistent symptomatic responses in a predictable time frame at a relevant pollen exposure level [12, 13]. Participants were administered their assigned treatment 2 h into the challenge. Nasal and ocular symptom severity was recorded by each participant at designated time points during the challenge. Symptom severity was rated on a scale of 0–3 (0, none; 1, mild; 2, moderate; 3, severe) (Table 1). Appropriate combinations of symptoms comprised the total nasal symptom score (TNSS), total ocular symptom score (TOSS), and total symptom score (TSS, an omnibus score comprised of all nasal and ocular symptoms) (Table 2).

**Table 1:** Rating scale for symptoms of seasonal allergic rhinitis

Score	Grade	Guideline
0	None	No sign/symptom is evident
1	Mild	Sign/symptom clearly present, but minimal awareness; easily tolerated
2	Moderate	Definite awareness of sign/symptom that is bothersome, but tolerable
3	Severe	Sign/symptom that is hard to tolerate; causes interference with activities during the challenge session

**Table 2:** Nasal and ocular symptoms of seasonal allergic rhinitis

Symptom	TNSS	TOSS	TSS
Runny nose (rhinorrhea)	X		X
Sneezing	X		X
Nasal itching	X		X
Itchy/red/gritty eye		X	X
Watery eyes		X	X

*TNSS* total nasal symptom score, *TOSS* total ocular symptom score, *TSS* total symptom score

Total scores were the sum of each individual symptom score (rated between 0 and 3); TNSS (0–9), TOSS (0–6), TSS (0–15)

### Study participants

Otherwise healthy male and female volunteers aged 18–65 years were included if they had a confirmed history of ragweed-induced SAR for the preceding two consecutive pollen seasons. A positive skin prick test response (defined as a wheal diameter ≥ 3 mm over diluent control) to ragweed pollen at screening or within 12 months of the screening visit was required. Full inclusion and exclusion criteria have been published elsewhere [10].

### Assessments

The primary endpoint of the present post hoc analysis was the change in TSS reported at each designated time point during the dosing periods in the study groups receiving loratadine or placebo. Secondary endpoints included the change in TOSS reported at each time point, and the percentage of participants who reported a symptom score of 0 or 1 (none or mild respectively) for all individual symptoms comprising the TSS or TOSS at each time point during the dosing periods.

### Statistical analyses

Similar statistical methodology used in the previous study was also employed in the current post hoc analysis to maintain experimental balance provided by the four-way crossover design. The per protocol (PP) population was included in the analysis and consisted of all participants who completed all four dosing periods in the original study. Efficacy analysis was performed at each post-baseline assessment time point for TSS and TOSS by using a mixed effects model with fixed effects for sequence, period, and treatment, and random effects for participant within sequence. Pairwise treatment comparisons with corresponding 95% confidence intervals were presented. Onset of action was evaluated based on the by-time point pairwise comparisons between loratadine and placebo obtained from the aforementioned model. The remaining secondary efficacy outcomes were analyzed using paired t tests and non-parametric equivalents. Statistical tests were performed at a nominal two-sided level of *P* = .05. No adjustments for multiplicity were made. All statistical analyses were performed using SAS statistical software, version 9.3 (NC, USA) and GraphPad Prism, version 6.0 (CA, USA).

## Results

### Participant demographics

A total of 70 participants were randomized into the study. Four participants did not complete all four dosing periods and were excluded from the PP population. A full report of participant demographics is provided in the original publication [10]. Briefly, the mean age (SD) was 35 (9.9) years and the majority of participants were Caucasian (97%) (Table 3). Nasal and ocular composite symptom scores were measured at baseline and summarized in Table 4.

**Table 3:** Summary of participant demographics

Characteristics	Overall (n = 66)
Mean age in years (SD)	35.0 (9.9)
Female (%)	39 (59)
Race (%)	
Caucasian	64 (97)
Black	0 (0)
Asian	2 (3)
American Indian/Alaska Native	0 (0)
Native Hawaiian/Other Pacific Islander	0 (0)
Other	0 (0)

**Table 4:** Baseline symptom scores

Baseline symptom scores (SD)^a^	Loratadine (n = 66)	Placebo (n = 66)
TNSS	6.9 (1.7)	6.5 (1.8)
TOSS	4.0 (1.5)	3.7 (1.7)
TSS	10.9 (2.6)	10.2 (3.0)

^a^Baseline symptom scores were collected immediately prior to dosing (i.e. 2 h after the start of allergen challenge on study day)

### Onset of action outcome

**Fig. 2:**
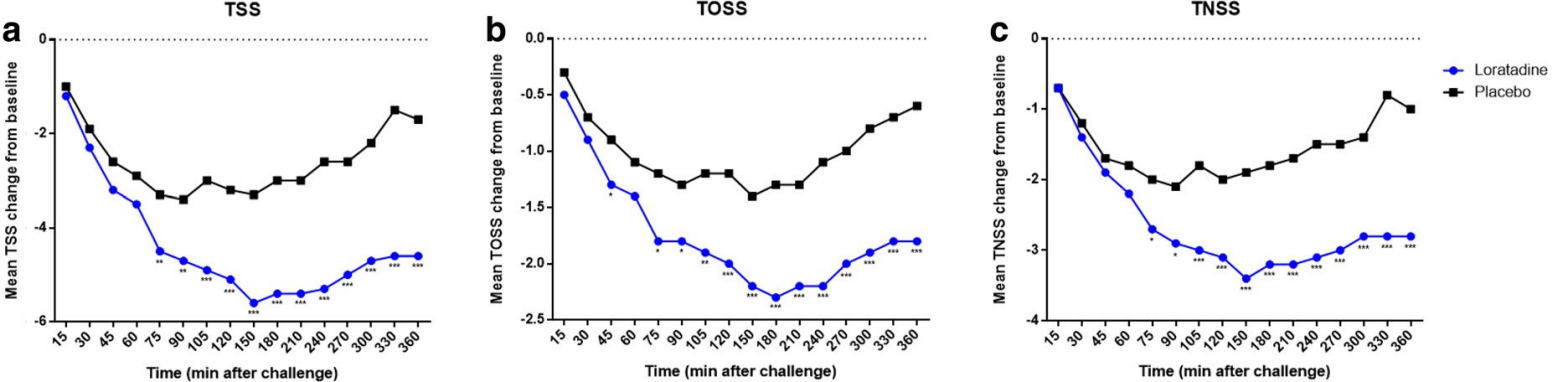
Change in nasal and ocular symptoms in loratadine and placebo groups during challenge. A significant improvement in TSS (**a**), TOSS (**b**), and TNSS (**c**) was observed at 75 min and remained significant for the remainder of the challenge period. *P ≤ .05, **P ≤ .01, ***P ≤ .001

Loratadine demonstrated a statistically significant improvement in TSS at 75 min post-treatment administration vs. placebo (*P* = .005). This improvement remained durable thereafter for the remainder of the allergen challenge (90 min, *P* = .003; all time points thereafter, *P* ≤ .001) (Fig. 2a; see also Additional file 1: Table S1 for exact *P* values).

### Secondary efficacy outcomes

A statistically significant improvement was first observed in TOSS at 45 min (*P* = .026) post-treatment, but did not remain durable until 75 min (*P* = .013) and thereafter (90 min, *P* = .022; 105 min, *P* = .002; all time points thereafter, *P* ≤ .001) (Fig. 2b; see also Additional file 2: Table S2 for exact *P* values). Similar improvements were observed in TNSS (statistics were previously reported by Ellis et al. [10]) (Fig. 2c).

**Fig. 3:**
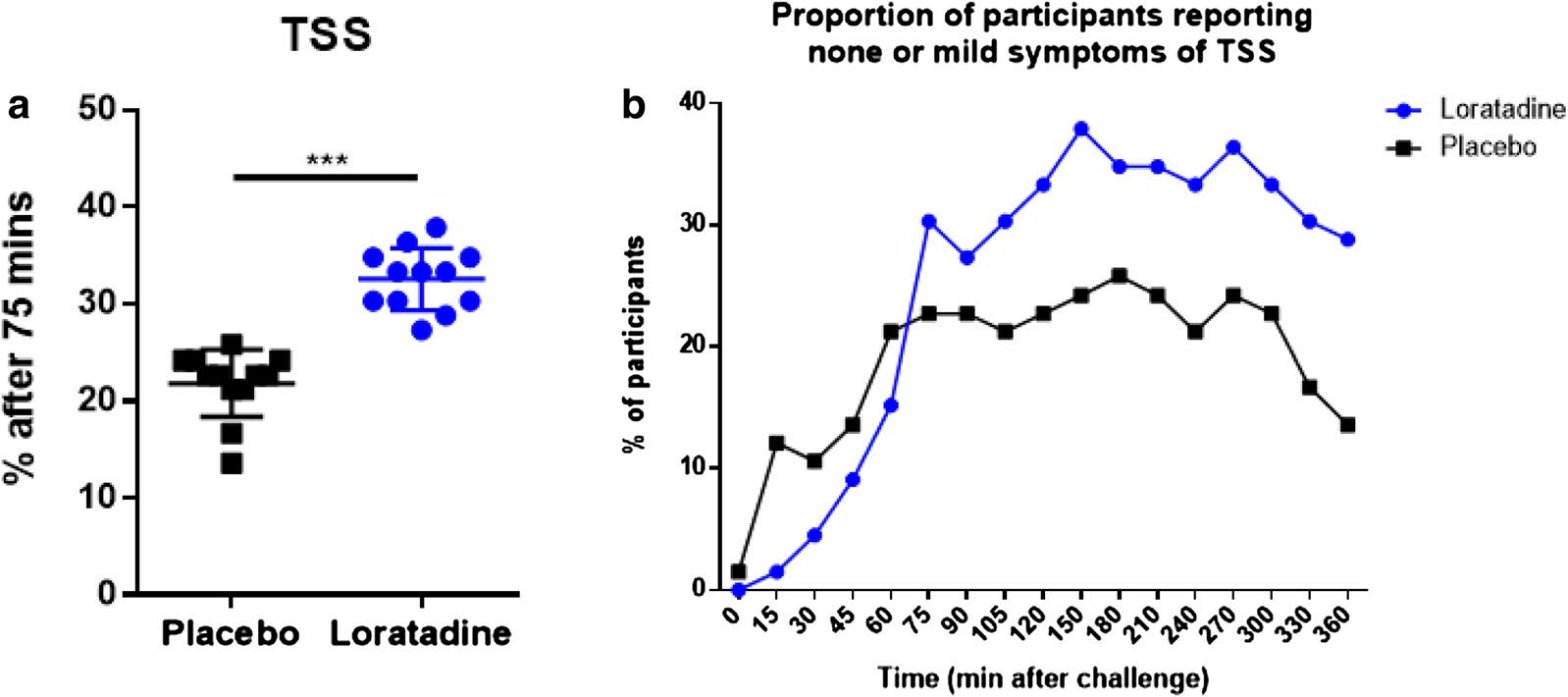
Proportion of participants reporting none or mild nasal and ocular symptoms during challenge. **a** Mean proportion of participants experiencing none or mild nasal and ocular symptoms was significantly greater in the loratadine group between 75 and 360 min. **b** Proportion of participants experiencing none or mild nasal and ocular symptoms remained higher in loratadine vs. placebo group at ≥ 75 min. Data are represented as mean ± SD (**a** only). ***P ≤ .001

A significantly higher mean proportion of participants receiving loratadine reported 0 or 1 for all component nasal and ocular symptoms comprising the TSS at 75 min and thereafter compared to participants receiving placebo. (32.6% vs. 21.8%, *P* = .0005) (Fig. 3a). This proportion was greater than placebo at 75 min, and remained greater for the duration of the challenge (Fig. 3b).

**Fig. 4:**
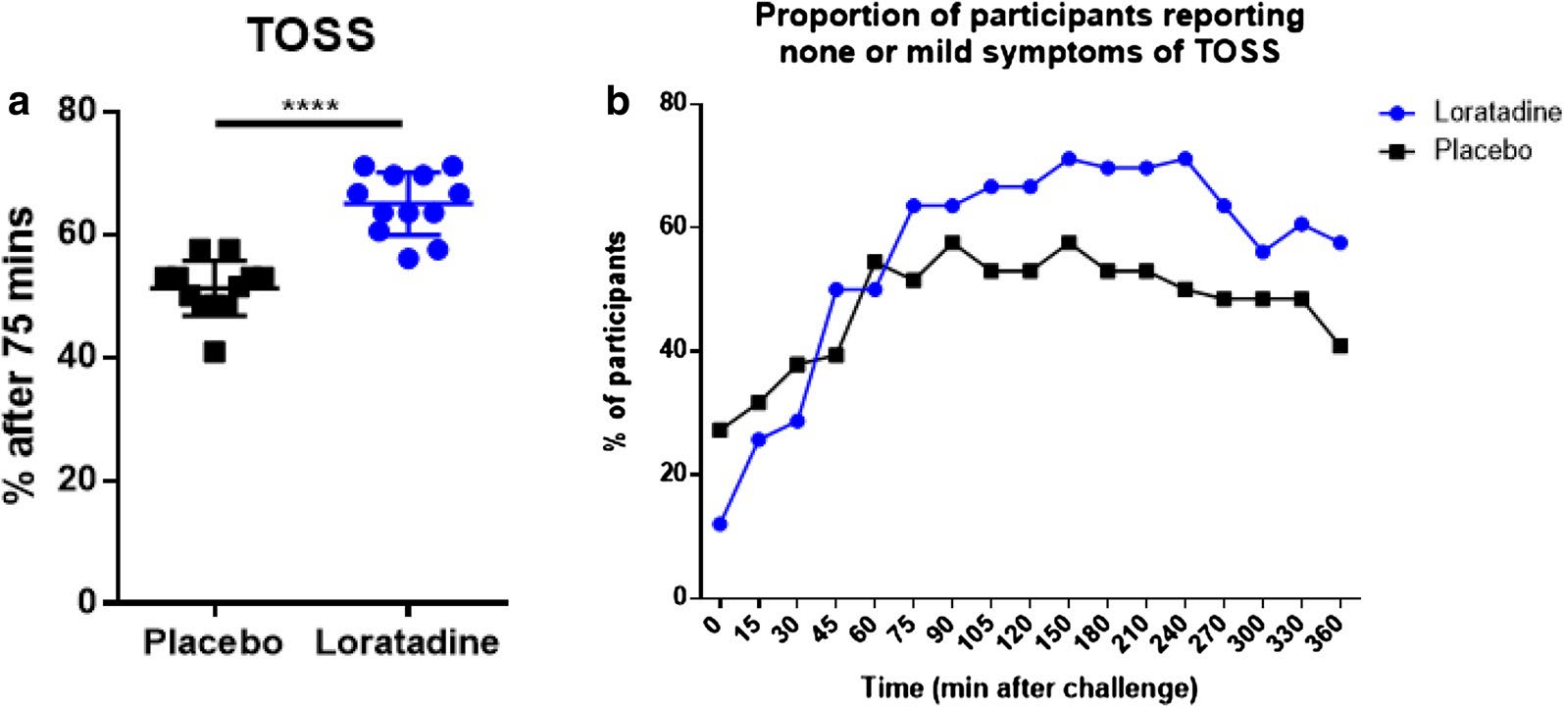
Proportion of participants reporting none or mild ocular symptoms during challenge. **a** Mean proportion of participants experiencing none or mild ocular symptoms was significantly greater in the loratadine group between 75 and 360 min. **b** Proportion of participants experiencing none or mild ocular symptoms remained higher in loratadine vs. placebo group at ≥ 75 min. Data are represented as mean ± SD (**a** only). ****P ≤ .0001

Similar findings were also observed for the two ocular symptoms of the TOSS composite in participants receiving loratadine vs. placebo (65.0% vs. 51.3%, *P* ≤ .0001) (Fig. 4a). This proportion was greater than placebo at 75 min, and remained greater for all time points thereafter (Fig. 4b).

### Safety

In this study, loratadine was well tolerated [10]. Sixty-eight and 69 participants received one dose of loratadine and placebo respectively. Serious adverse events or deaths were not reported during the dosing periods. A total of 12 and 5 adverse events (AEs) were reported in the loratadine (4 mild and 8 moderate) and placebo (2 mild and 3 moderate) groups respectively. Only one report of mild urticaria was considered possibly related to the study medication (loratadine) (Table 5).

**Table 5:** Characteristics of treatment emergent adverse events. Adapted with permission from © Ellis et al. [10]

	Loratadine (n = 68)	Placebo (n = 69)
Number of participants reporting ≥ 1 AEs (%)	7 (10)	4 (6)
Number of AEs reported	12	5
Serious (%)		
No	12 (100)	5 (100)
Yes	0 (0)	0 (0)
Severity of AE (%)		
Mild	4 (33)	2 (40)
Moderate	8 (67)	3 (60)
Severe	0 (0)	0 (0)
Possible relationship to study medication (%)		
Not possibly related	11 (92)	5 (100)
Possibly related	1 (8)	0 (0)

## Discussion

The current post hoc analysis evaluated the onset of total symptom relief related to unmodified loratadine tablets. Results demonstrated an onset of action of 75 min for the relief of both nasal and ocular symptoms in SAR participants. These results contrast the longer onset of action (180 min) observed for loratadine previously [12, 13]. However, these earlier studies evaluated an encapsulated formulation of loratadine (loratadine capsules).

According to in vitro dissolution studies, loratadine is classified as a Biopharmaceutics Classification System (BCS) Class II drug [20]. BCS Class II drugs have low solubility and high permeability, making it difficult to accurately evaluate the bioavailability of different formulations and bioequivalence among immediate release solid oral dosage forms [21]. It follows that bioequivalence cannot be assumed for loratadine tablets and gelatin-encapsulated capsules, unless demonstrated by appropriate bioequivalence studies.

Apart from the aforementioned differences in the onset of action, both loratadine tablets and gelatin-encapsulated capsules are able to provide symptomatic relief of SAR-associated symptoms [10, 12–14]. In the current analysis, approximately 30.3% of participants in the loratadine arm experienced none or mild nasal/ocular symptoms at product onset, 75 min after dose administration. A higher level of satisfaction was reported in the earlier EEU study conducted using loratadine capsules, where 53.5% of participants reported experiencing major or moderate improvement in allergy symptoms (captured as global efficacy responses) at the end of the study [13]. Both EEU studies demonstrated a significant improvement in the loratadine arms (tablets or capsules) over placebo [10, 13].

Consistent with other published in-season studies, the efficacy of loratadine capsules in patients with SAR symptoms has been demonstrated. For example, treatment with loratadine capsule once daily for four weeks led to an overall improvement in ocular symptoms by week 1, and total symptom scores over the treatment period [22, 23]. Thus, encapsulated loratadine is efficacious in providing symptomatic relief for SAR patients. Since there are no head-to-head EEU studies comparing the onset of the two dosage forms (tablet vs. capsule), the onset of the loratadine capsule is likely different from the unmodified loratadine tablet.

## Conclusions

The current post hoc analysis demonstrated an onset of action of 75 min for unmodified loratadine tablets. The longer onset of action previously reported by Day et al. is most likely attributed to a delayed release of loratadine from an over-encapsulated tablet that was evaluated in the study. As bioequivalence cannot be assumed between loratadine dosage forms, and since the active is a BCS Class II drug, one must be mindful when interpreting onset data generated with dosage forms that have been altered from their manufactured form.

## Additional files

**Additional file 1: Table S1.** Change from baseline in Total Symptom Score (TSS) in loratadine and placebo groups.

**Additional file 2: Table S2.** Change from baseline in Total Ocular Symptom Score (TOSS) in loratadine and placebo groups.

## Abbreviations

AR: allergic rhinitis
BCS: Biopharmaceutics Classification System
EEU: Environmental Exposure Unit
SAR: seasonal allergic rhinitis
TNSS: total nasal symptom score
TOSS: total ocular symptom score
TSS: total symptom score
PP: per protocol
