# Supplementary material for: Onset of action for loratadine tablets for the symptomatic control of seasonal allergic rhinitis in adults challenged with ragweed pollen in the Environmental Exposure Unit: a post hoc analysis of total symptom score
Source: Allergy Asthma Clin Immunol. 2018 Jan 16;14:5. doi: 10.1186/s13223-017-0227-4 (PMC5771028; doi:10.1186/s13223-017-0227-4)
Supplement: Supplementary file 1 — Additional file 1: Table S1. Change from baseline in Total Symptom Score (TSS) in loratadine and placebo groups. [file 13223_2017_227_MOESM1_ESM.docx]

| Post-Baseline Time Point | Study Treatment | | | | Pairwise Treatment Comparisons**^†^** | | | |
| --- | --- | --- | --- | --- | --- | --- | --- | --- |
|  | Loratadine (n=66) | | Placebo (n=66) | |  |  |  |  |
|  | Mean***** | S.D. | Mean***** | S.D. | Difference**^††^** | Lower 95% CI | Upper 95% CI | p-value |
| 15 Min. | -1.2 | 1.79 | -1.0 | 2.00 | -0.2 | -0.9 | 0.4 | 0.483 |
| 30 Min. | -2.3 | 2.53 | -1.9 | 3.16 | -0.4 | -1.1 | 0.4 | 0.333 |
| 45 Min. | -3.2 | 2.83 | -2.6 | 3.11 | -0.6 | -1.3 | 0.2 | 0.124 |
| 60 Min. | -3.5 | 3.13 | -2.9 | 3.28 | -0.6 | -1.5 | 0.3 | 0.168 |
| 75 Min. | -4.5 | 3.37 | -3.3 | 3.14 | -1.2 | -2.0 | -0.4 | 0.005 |
| 90 Min. | -4.7 | 3.36 | -3.4 | 3.36 | -1.3 | -2.1 | -0.4 | 0.003 |
| 105 Min. | -4.9 | 3.29 | -3.0 | 3.54 | -1.9 | -2.8 | -1.1 | < .001 |
| 120 Min. | -5.1 | 3.60 | -3.2 | 3.33 | -1.8 | -2.7 | -1.0 | < .001 |
| 150 Min. | -5.6 | 3.61 | -3.3 | 3.54 | -2.2 | -3.1 | -1.3 | < .001 |
| 180 Min. | -5.4 | 3.67 | -3.0 | 3.54 | -2.3 | -3.2 | -1.4 | < .001 |
| 210 Min. | -5.4 | 3.73 | -3.0 | 3.60 | -2.3 | -3.2 | -1.4 | < .001 |
| 240 Min. | -5.3 | 3.65 | -2.6 | 3.74 | -2.7 | -3.6 | -1.7 | < .001 |
| 270 Min. | -5.0 | 3.71 | -2.6 | 3.59 | -2.4 | -3.4 | -1.4 | < .001 |
| 300 Min. | -4.7 | 3.91 | -2.2 | 3.56 | -2.4 | -3.4 | -1.5 | < .001 |
| 330 Min. | -4.6 | 3.64 | -1.5 | 3.60 | -3.1 | -4.0 | -2.1 | < .001 |
| 360 Min. | -4.6 | 3.62 | -1.7 | 3.49 | -2.9 | -3.9 | -2.0 | < .001 |

**Table S1.** Change from baseline in Total Symptom Score (TSS) in loratadine and placebo groups.

SD, standard deviation; CI, confidence interval.

**^*^**Means were calculated as post-baseline measurement subtract baseline measurement. Negative values indicate symptom improvement.

**^†^**Pairwise treatment comparisons of loratadine versus placebo were based on analysis of full data set from the four-period cross-over study.

**^††^**Differences were calculated as loratadine subtract placebo. Negative values directionally favor loratadine.
