# Supplementary material for: Onset of action for loratadine tablets for the symptomatic control of seasonal allergic rhinitis in adults challenged with ragweed pollen in the Environmental Exposure Unit: a post hoc analysis of total symptom score
Source: Allergy Asthma Clin Immunol. 2018 Jan 16;14:5. doi: 10.1186/s13223-017-0227-4 (PMC5771028; doi:10.1186/s13223-017-0227-4)
Supplement: Supplementary file 2 — Additional file 2: Table S2. Change from baseline in Total Ocular Symptom Score (TOSS) in loratadine and placebo groups. [file 13223_2017_227_MOESM2_ESM.docx]

| Post-Baseline Time Point | Study Treatment | | | | Pairwise Treatment Comparisons**^†^** | | | |
| --- | --- | --- | --- | --- | --- | --- | --- | --- |
|  | Loratadine (n=66) | | Placebo (n=66) | |  |  |  |  |
|  | Mean***** | S.D. | Mean***** | S.D. | Difference**^††^** | Lower 95% CI | Upper 95% CI | p-value |
| 15 Min. | -0.5 | 0.85 | -0.3 | 0.94 | -0.2 | -0.5 | 0.1 | 0.129 |
| 30 Min. | -0.9 | 1.14 | -0.7 | 1.54 | -0.2 | -0.6 | 0.1 | 0.222 |
| 45 Min. | -1.3 | 1.36 | -0.9 | 1.42 | -0.4 | -0.8 | -0.1 | 0.026 |
| 60 Min. | -1.4 | 1.44 | -1.1 | 1.43 | -0.2 | -0.7 | 0.2 | 0.238 |
| 75 Min. | -1.8 | 1.52 | -1.2 | 1.54 | -0.5 | -1.0 | -0.1 | 0.013 |
| 90 Min. | -1.8 | 1.57 | -1.3 | 1.64 | -0.5 | -0.9 | -0.1 | 0.022 |
| 105 Min. | -1.9 | 1.52 | -1.2 | 1.65 | -0.7 | -1.1 | -0.3 | 0.002 |
| 120 Min. | -2.0 | 1.55 | -1.2 | 1.58 | -0.8 | -1.3 | -0.4 | < .001 |
| 150 Min. | -2.2 | 1.64 | -1.4 | 1.63 | -0.8 | -1.2 | -0.3 | < .001 |
| 180 Min. | -2.3 | 1.60 | -1.3 | 1.64 | -1.0 | -1.4 | -0.5 | < .001 |
| 210 Min. | -2.2 | 1.59 | -1.3 | 1.65 | -0.9 | -1.4 | -0.5 | < .001 |
| 240 Min. | -2.2 | 1.56 | -1.1 | 1.81 | -1.1 | -1.5 | -0.6 | < .001 |
| 270 Min. | -2.0 | 1.67 | -1.0 | 1.74 | -1.0 | -1.4 | -0.5 | < .001 |
| 300 Min. | -1.9 | 1.63 | -0.8 | 1.64 | -1.0 | -1.5 | -0.6 | < .001 |
| 330 Min. | -1.8 | 1.58 | -0.7 | 1.78 | -1.1 | -1.6 | -0.6 | < .001 |
| 360 Min. | -1.8 | 1.54 | -0.6 | 1.67 | -1.2 | -1.7 | -0.8 | < .001 |

**Table S2.** Change from baseline in Total Ocular Symptom Score (TOSS) in loratadine and placebo groups.

SD, standard deviation; CI, confidence interval.

**^*^**Means were calculated as post-baseline measurement subtract baseline measurement. Negative values indicate symptom improvement.

**^†^**Pairwise treatment comparisons of loratadine versus placebo were based on analysis of full data set from the four-period cross-over study.

**^††^**Differences were calculated as loratadine subtract placebo. Negative values directionally favor loratadine.
